# Effect of Laser Acupuncture on Disuse Osteoarthritis: An Ultrasound Biomicroscopic Study of Patellar Articular Cartilage in Rats

**DOI:** 10.1155/2012/838420

**Published:** 2012-07-19

**Authors:** Qing Wang, Xia Guo, Mu-Qing Liu, Xiao-Yun Wang, Yong-Ping Zheng

**Affiliations:** ^1^Department of Rehabilitation Sciences, The Hong Kong Polytechnic University, Hong Kong; ^2^Institute of Medical Information, School of Biomedical Engineering, Southern Medical University, Guangzhou, China; ^3^Department of Hand Surgery, Tsinghua University, Yuquan Hospital, Beijing 100049, China; ^4^Interdisciplinary Division of Biomedical Engineering, The Hong Kong Polytechnic University, Hong Kong

## Abstract

To investigate the effect of laser acupuncture (LA) on disuse changes in articular cartilage using ultrasound biomicroscopy (UBM), Eighteen rats were randomly divided into the control group (C), the tail-suspended group (T), and the tail-suspended with LA treatment group (L). During 28-day suspension period, group L were treated with LA at acupoints on the left hindlimb while group T had a sham treatment. Ultrasound roughness index (URI), integrated reflection coefficient (IRC), integrated backscatter coefficient (IBC), cartilage thickness, and ultrasonographic score (US) of articular cartilage at patella were measured by using an ultrasound biomicroscopy system (UBS). Compared with the group C, URI significantly (*P* < 0.01) increased by 60.9% in group T, increased by 38.1% in group L. In addition, unloading induced a significant cartilage thinning (*P* < 0.05) in group T, whereas cartilage thickness in group L was 140.22 ± 19.61 **μ**m reaching the level of the control group (147.00 ± 23.99 **μ**m). There was no significant difference in IRC, IBC, and US among the three groups. LA therapy could help to retain the quality of articular cartilage which was subjected to unloading. LA would be a simple and safe nonpharmacological countermeasure for unloading-induced osteoarthritis. The UBM system has potential to be a sensitive, specific tool for quantitative assessment of articular cartilage.

## 1. Introduction

Articular cartilage use can be distinguished in to 3 different patterns that have different consequences: normal use, marked decrease use (disuse), and use that damages the tissue (abuse). Patellar articular cartilage is subjected to high loads under a ground reaction force during daily walking, running, and jumping, which are important for keeping knees healthy. Both *in vitro* and *in vivo* experimental models demonstrate that articular cartilage can respond to both increased or decreased loading [1]. It has been found that the absence of joint loading associates with markedly increased percentage of apoptotic chondrocytes [2], decrease of matrix synthesis and content production and consequently cartilage thinning [3–5]. These site-specific changes of articular cartilage could be a reversible tentative phenomenon or progress to osteoarthritis (OA), depending on the magnitude and type of altered loading [6].

The apoptosis of chondrocytes and synthesis of matrix can be influenced by intrinsic or extrinsic factors in addition to mechanical loading. Acupuncture is an ancient Chinese method of healing. Previous studies have shown the efficiency of acupuncture on prevention of postmenopausal osteoporosis in humans [7] and in ovariectomized rats [8, 9]. Laser acupuncture (LA) is an noninvasive alternative too and has been proved equal biological effect as needle acupuncture [10]. LA has been shown as an effective countermeasure for unloading-induced bone loss [11]. It is unknown whether it can subside cartilage degeneration caused by unloading. 

High-frequency ultrasound biomicroscopy (UBM) system has been developed for clear imaging of tiny tissues such as articular cartilage [12–17] and cornea [18]. Earlier studies on ultrasonic characteristics of articular cartilage have used echogenicity, sound speed, reflection coefficient, backscatter coefficient, attenuation, and ultrasound roughness index (URI) to reflect the structural and constituent modifications of the tissue. In comparison with traditional musculoskeletal ultrasound imaging (5–12 MHz) applied to monitor microgravity-induced changes in muscles, tendons, and bone [19], high-frequency UBM providing a resolution of approximately 30 *μ*m or even higher has potentials to be a highly sensitive, specific tool for quantitative analysis of microstructure of tiny tissues such as articular cartilage [12, 16, 20, 21], intervertebral discs [22], and cornea [18].

The tail suspension rat models [17, 23] have been used to investigate space flight-like effects [24] on the body. The aim of this study was to investigate the countermeasure effect of LA on the unloading-induced changes in patellar articular cartilage in tail-suspension rat model using UBM.

## 2. Materials and Methods

### 2.1. Animal Care and Experimental Protocol

**Figure 1:**
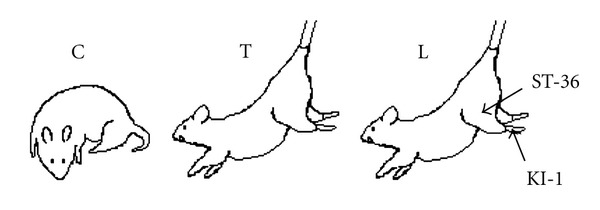
Schematic of the experiment protocol: rats in group C under normal gravity, rats in group T suspended in cage by wrapping the tail with an angle of 30° between the rat body and the cage floor, and rats in group L suspended as rats in group T and daily treated by laser acupuncture at Zu San Li (ST-36) and Yong Quang (KI-1) acupoints.

Eighteen Sprague-Dawley rats (male, 3-month old, body weight 400 ± 20 g) were divided randomly into 3 groups (*n* = 6 for each group): the control group (C), the tail suspended group (T), and the tail-suspended with laser acupuncture treatment group (L). The animal care and the protocol of the tail suspension and the LA treatment were similar to our previous studies [11, 17]. Rats in groups T and L underwent 28-day tail suspension. Rats were suspended with a tail harness that raised their hindlimbs and kept them in a head-down position. The angle formed between the body of the rat and the floor of the cage was 30° [22]. The rats in group C were under normal activity (Figure 1). Animals were kept individually in metal cages and fed with standard rabbit diet and water ad lib. Ethics approval was obtained from the Animal Ethics Committee of the administering institution in Hong Kong before conducting the experiment.

In group L, LA therapy was performed under general anesthesia with intraperitoneal injection of 5% chloral hydrate (1 mL/100 g) and with the rat in the supine position. Acupoints including Zu San Li (ST-36 is located at the belly of the tibialis cranialis muscle below the cranial crest of the tibia) and Yong Quan (KI-1, at the plantar depression in the middle of the metatarsal area of the foot) of the left leg were selected in this study [11]. LA treatment was provided daily for 28 consecutive days by placing an LA probe (Ito Laser, Lhasa Medical, Weymouth, MA) at ST-36 and KI-1 acupoints and providing LA stimulation for 3 minutes. The laser parameters were 5 mW, wavelength of 670 nm, and a beam spot size of 19.6 mm^2^. Rats in group T underwent a sham treatment which is the same procedure as in group L except that the laser was not turned on. Rats in group C received general anesthesia daily as same as rats in groups T and L without any treatment. On day 29, all the animals were euthanized with an overdose of sodium pentobarbital (Euthanyl, MTC Pharmaceutical, Cambridge, Ontario, Canada). Patellae were excised, wrapped in wet gauze soaked with physiological saline, and stored at −20°C until ultrasound examination.

### 2.2. UBM System

**Figure 2:**
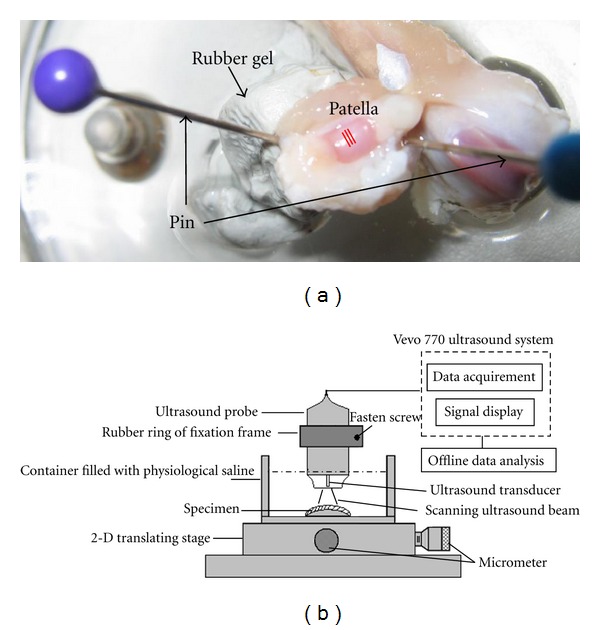
(a) A picture of a typical patellar specimen fixed in the container. Three dashed lines present the locations of three ultrasound scanning lines in the central of the patella. (b) Schematic of the UBM system used to assess acoustic parameters and cartilage thickness.

The UBM system used in this study includes a 2-dimentional (2D) translating stage (Model 2201MMXY, Ball Slide Positioning Stages, Del-Tron Precision Inc., Bethel, CT, USA), a high-resolution ultrasound imaging system specially developed for small animal research (Vevo 770, Visual-Sonics Inc., Toronto, ON, Canada), and a custom-designed MATLAB program (The Math-Works, Natick, MA, USA) for the offline extraction of ultrasonic parameters (Figure 2(b)). The high-frequency ultrasound probe (RMV-708) is composed by a focused transducer with a nominal central frequency of 55 MHz and a focal length of 4.5 mm. The axial resolution is approximately 30 *μ*m. The transducer is performed with a mechanical-sector scan in real time. 2-D UBM images were acquired at a frame rate of 20 fps in this study. 

Before ultrasound examination, the specimen was thawed in the physiological saline solution. The container was fixed on the 2-D translating stage. A block of the rubber gel (Blu-Tack, Thomastown, Australia) was attached on the container bottom. The patella sample was fixed on the rubber gel by inserting two pins into the surrounding soft tissues and the rubber gel, as shown in Figure 2(a). Then the container was filled fully with the physiological saline solution. The ultrasound probe was installed on a fixation frame and placed above the cartilage surface with the perpendicular incidence of ultrasound beam and with the focal zone of the ultrasound beam in the middle of the tissue by adjusting the slider. The ultrasound scanning was performed for three times with an interval of 200 *μ*m between two scanning lines. The interval value was adjusted using the micrometers on the 2-D translating stage. UBM images were recorded for offline data analysis of parameter extraction.

### 2.3. Quantitative Parameters

**Figure 3:**
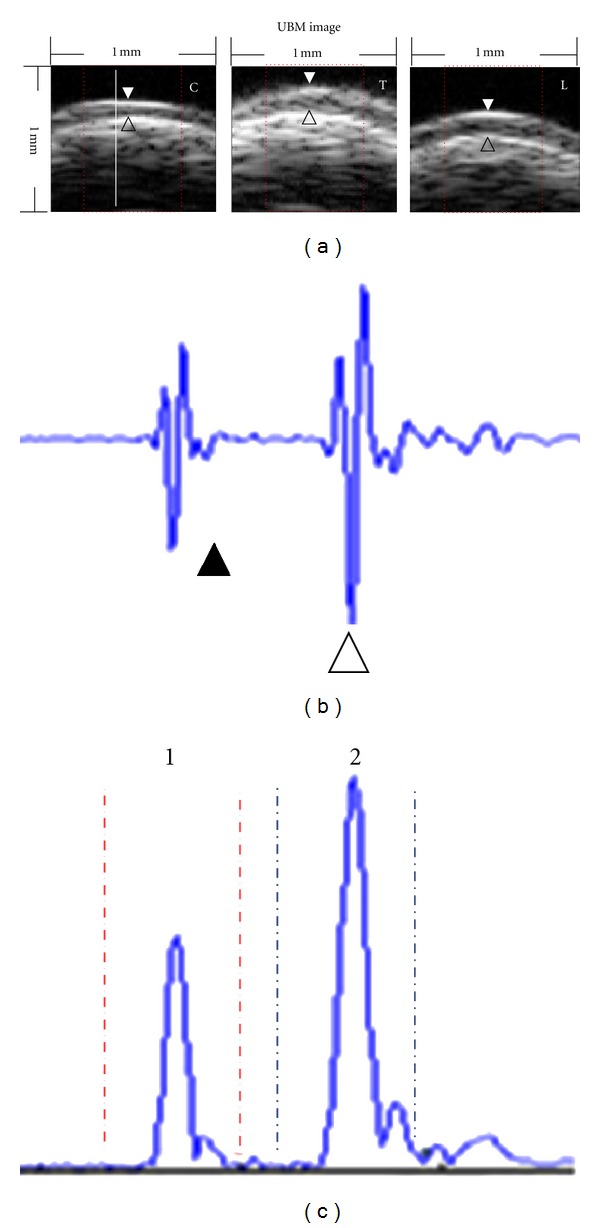
(a) The typical UBM images of patella in groups C, T, and L. White arrowhead and black open arrowhead indicate the cartilage surface and the cartilage-bone interface, respectively. (b) One typical scan line (from the white dashed line in (a)) of ultrasound RF signal reflected from the cartilage surface (▲) and the cartilage-bone interface (∆). (c) The Hilbert envelope signal of the RF signal in (b). Two windows were set to locate the position of the cartilage surface and the cartilage-bone interface. “1” and “2” indicate window 1 and window 2, respectively.

A region of interest (ROI) (1 mm × 1 mm) was selected in the transverse UBM image and included 100 lines of ultrasonic radiofrequency (RF) data. In view of the severe curvature of the cartilage surface at patella, only three-fifths (i.e., 60 sampling lines) of the central ROI were used for calculation (Figure 3(a)). To locate the cartilage surface and the cartilage-bone interface, the ultrasonic RF data of each sampling line (Figure 3(b)) were processed using Hilbert transform.

The peak points with the maximum value of the Hilbert envelope data were, respectively, searched in two manually set windows (Figure 3(c)). The cartilage surface was located at the peak point in the first window, while the cartilage-bone interface was located at the peak point in the second window. Then, the contour profiles of the cartilage surface and the cartilage-bone interface were drawn along the first peak points and the second peak points in 60 sampling lines, respectively. The distance from the transducer to the cartilage surface (*d*_*i*1_) and the distance from the transducer to the cartilage-bone interface (*d*_*i*2_) along the *i*th sampling line were recorded (*i* = 16, 17, …, 75). Then the averaged thickness of the cartilage tissue was calculated:

(1) $$\begin{array}{c} \text{Thickness}=\frac{1}{m}\sum_{i}\left(d_{i1}-d_{i2}\right), \end{array}$$

where *m* is the total number of the sampling lines, equal to 60 in this study.

Ultrasound roughness index (URI) was defined to indicate the roughness or smoothness of the cartilage surface. In consideration with the natural curvature of the cartilage surface in a relatively low-frequency range and the true surface roughness rather in a relatively high-frequency range, the curvature of the surface contour was eliminated using high-pass filter before the URI calculation [15]. Then, URI was determined from the filtered profile of the cartilage surface:

(2) $$\begin{array}{c} \text{Roughness}=\sqrt{\frac{1}{m}\sum_{i}{\left(d_{i}-\overset{-}{d}\right)}^{2},} \end{array}$$

where *d*_*i*_ is the distance from the transducer to the cartilage surface in the *i*th sampling line and $\overset{-}{d}$ is the averaged value of *d*_*i*_ (*i* = 16, 17, …, 75).

This study defined integrated reflection coefficient (IRC) and integrated backscatter coefficient (IBC) to evaluate the acoustic property of the cartilage surface and the cartilage-bone interface, respectively. In this study, the power spectrum of the signals reflected from the cartilage surface was calibrated by a reference spectrum of the signals reflected from a steel plate in physiological saline. Similarly, the power spectrum of the signals reflected from the cartilage-bone surface was processed with the same calibration. IRC and IBC were, respectively, calculated using [25]

(3) $$\begin{array}{c} \text{IRC}=\frac{1}{f_{2}-f_{1}}\int_{f_{1}}^{f_{2}}B_{1}\left(f\right)df,\\ \text{IBC}=\frac{1}{f_{2}-f_{1}}\int_{f_{1}}^{f_{2}}B_{2}\left(f\right)df, \end{array}$$

where *f*_1_ = 18 MHz and *f*_2_ = 55 MHz are the lower and upper limits of the −3 dB bandwidth of the transducer, respectively. *B*_1_(*f*) is the backscatter transfer function of the calibrated spectrum of the cartilage surface. *B*_2_(*f*) is the backscatter transfer function of the calibrated spectrum of the cartilage-bone interface.

### 2.4. Semiquantitative Evaluation

Semiquantitative evaluation of the UBM images was performed according to an ultrasonographic grading system scoring on a scale of 1–6, where 1 is the sharp and bright superficial interface; 2 is the less bright and/or less sharp superficial interface; 3 is the irregular superficial interface with full loss of brightness or small superficial cleft; 4 is the partial cartilage thickness defect; 5 is the focal full cartilage thickness defect; 6 is the loss of full cartilage thickness with deep interface irregularity. This ultrasonographic grading system is revised in accordance with Spriet's study [16]. The ultrasonographic grade was scored blindly and independently by three researchers. All three scans of the patellar cartilage were reviewed and the highest grade was attributed to the cartilage tissue. The grades given by the three researchers were ranked and the middle one was the final score for the sample.

### 2.5. Statistical Analysis

Statistical analyses were conducted with SPSS software (V17, SPSS Inc., Chicago, USA). All values in the text are presented as mean ± standard deviation (SD). The statistical differences in the thickness, URI, IRC, and IBC between groups were analyzed using one-way ANOVA and LSD post hoc tests. A paired *t*-test was used to compare these UBM extracted values between the left and right patella of rats in the group L. Statistical significance was considered when *P* < 0.05.

## 3. Results

**Figure 4:**
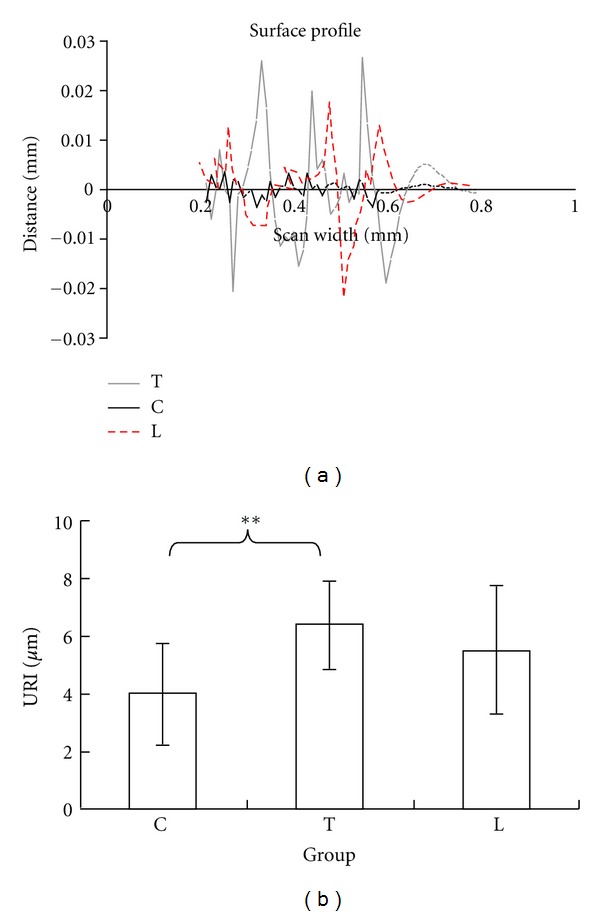
(a) The surface profile of the cartilage surface extracted from ROI (red dashed rectangle in Figure 3(a)). (b) URI of groups C, T, and L. **Statistically significant difference at level *P* < 0.01.

Mean values (±SD) and percentage values of all the parameters are presented in Table 1. As shown in Figure 4(a), the contour profile extracted from the cartilage surface of the specimen in group T is the most fluctuant among the three groups. One-way ANOVA showed the difference in URI was significant (*P* = 0.015). Post-hoc analysis indicated that the roughness of group T increased significantly (*P* = 0.004) by 60.9% (Figure 4(b)) after 28-day unloading in comparison with group C, whereas the roughness of group L increased insignificantly (*P* = 0.075) by 38.1%. There was no significant difference in URI between groups T and L (*P* = 0.270). 

Though ANOVA showed no significant difference (*P* = 0.115) in the thickness of cartilage among 3 groups, post-hoc analysis showed significant decrease (*P* = 0.043) in the thickness of cartilage by 13.6% in group T compared with group C, whereas the thickness of articular cartilage in group L decreased only 4.6% which was a insignificant change (*P* = 0.489) compared with group C.

ANOVA showed no significant differences among groups in IRC (*P* = 0.463) which reflected the acoustic property of the cartilage surface and IBC (*P* = 0.217) which reflected the acoustic property of the cartilage-bone interface.

**Figure 5:**
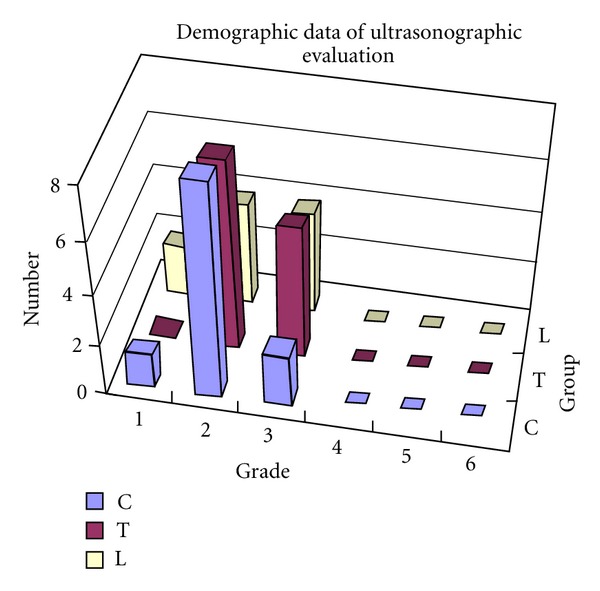
Demographic result of ultrasonographic evaluation on the patellar cartilage in groups C, T, and L.

Semiquantitative evaluation showed that most samples in all three groups were without macroscopic lesions and were graded either 1 or 2 of 6 grades distinguished at ultrasonographic evaluation (Figure 5) while grade 3 was considered as the first evident pathological grade (Table 1).

Paired *t *tests showed no significant difference in the UBM extracted values between the left and right patella of rats in L group, although the LA was applied only to the acupoints located on the left leg.

## 4. Discussion

The quantitative results indicate that the unloading of the 28-day tail suspension induced changes in both surface roughness and thickness of patellar articular cartilage and the LA stimulation to ST-36 and KI-1 acupoints could subside these changes.

Our previous study found that 28-day unloading affected articular cartilage site-dependently in ultrasound parameters [17]. Relatively severe alterations were found in patella including cartilage thinning and the increase of surface roughness. Hence, this study selected patellar articular cartilage as study samples. The therapeutic effects of LA on patellar articular cartilage subjected to unloading were assessed by using a high-frequency UBM system. Comparing the diagnostic ability of different ultrasound parameters, the coefficients related to ultrasound reflection or backscatter tend to be sensitive for the investigation of cartilage matrix structural changes [12], whereas URI is a recently introduced parameter of assessing the surface irregularity of the cartilage tissue [15, 26]. Another ultrasound parameter is attenuation that could reflect more information on the tissue composition (PG, collagen, or water content) and mechanical properties (stiffness) [27]. Based on those previous studies, the acoustic parameters correlated with the morphologic and mechanical properties of the degraded cartilage tissue were investigated in recent studies [13, 17, 28, 29]. This study therefore chose URI, IRC, IBC, cartilage thickness, and ultrasonographic grade as the parameters to evaluate the effect of unloading and the corresponding countermeasure effect of LA on patellar cartilage. Using UBM, the impact of disuse on the surface morphology (surface roughness measured by URI) and the apparent morphology (cartilage thickness) are clearly demonstrated, whereas the impact on the composition changes measured by the reflection coefficients (IRC, IBC) of both the cartilage surface and the cartilage-bone interface was not significant. The cartilage thickness and URI of the cartilage surface could be more sensitive indexes to indicate the disuse changes in articular cartilage than IRC, IBC. In addition, the quantitative analysis of the morphology parameters (cartilage thickness and surface roughness) might be more sensitive than the semiquantitative evaluation of the UBM images (sonographic grade).

The UBM finding for changes in the thickness and URI of patellar cartilage supports findings of previous studies [17, 30–32]. Following LA treatment there was a significant lower value in URI and a higher value in cartilage thickness compared with group T (Table 1). The results indicated the retaining of ultrasonic characteristics of patellar articular cartilage by daily LA treatment. The exact mechanism of LA effect cannot be answered by this study. However, to eliminate the local effect of LA, LA treatment was only applied to the left hindlimb while results were examined in both patellae. The results showed no significant difference between the left and right sides. This suggests that LA retains ultrasonic characteristics of patellar articular cartilage through a systemic regulation rather than a local effect.

In light of traditional Chinese medicine (TCM), life is governed by the interaction of *Yin* and *Yang* and *Qi* is the vital energy flowing through the meridians to keep *Yin-Yang* in balance. When *Yin* and *Yang* are in imbalance and *Qi* is obstructed, it results in diseases. In an ancient TCM book, *Inner Classic of the Yellow Emperor*, which was written around third century B.C., needle acupuncture was already mentioned as an important therapy to rebalance *Yin-Yang* and circulate *Qi* clearly in the meridians. With the most recent technologic development, LA has been introduced. Without insertion into the skin, the laser probe is placed at acupoints to generate the low-intensity, nonthermal laser irradiation. It is found that the therapeutic efficacy of LA depends on the depth of laser energy transmission [33]. Some studies demonstrated the similarity between LA stimulation and the stimulation of traditional needle acupuncture or electroacupuncture (EA). Litscher [10] reported that the LA-generated light stimulus was analogous to mechanical pressure by manipulating the needle. Komori et al. [34] provided evidence that both needle acupuncture and LA therapy had similar effects on microcirculation. Hsieh et al. [35] pointed out that the frequency-dependent brain-area activation stimulated by LA was similar to that induced by electroacupuncture (EA). With the worldwide training and provision of acupuncture care in the recent two decades, an increasing attention has been paid to the scientific evaluation of acupuncture treatment of various diseases including OA pain [36, 37]. 

However, few studies have reported on LA countermeasure of cartilage degeneration. The present study selected KI-1 and ST-36 as treatment sites and found the therapeutic effect of LA on the acoustic and morphological properties of patellar articular cartilage subjected to unloading. KI-1 (Yong Quan-Bubbling Spring) is the first acupoint of the Kidney meridian, where life begins to gush, coiling up through the body. The *Inner Classic of the Yellow Emperor* notes that kidney strength manifests in strong bones. KI-1 acupuncture strengthens kidney function. Hsieh et al. [35] conducted a study to investigate the possible mechanism of LA at KI-1. Their results demonstrated that its mechanism concerns the nervous system (afferent sensory information processing) and the circulatory system (hemodynamic properties). The Stomach takes *Yang* (taking in food) and also shows *Yin* (assimilation and nourishment). Thus the Stomach channel is important for balancing *Yin-Yang*. ST-36 (Zu San Li) is a key point of the Stomach meridian and frequently used in acupuncture therapy. For example, Zhang et al. [8] needled at ST-36 and SP-6 (San Yin Jiao) to effectively prevent bone loss in ovariectomized rats and Guo et al. [11] stimulated ST-36 and KI-1 to improve intestinal calcium absorption to counter the inhibitory effect of microgravity on bones [38]. It was also found by Yin et al. [39] that glycometabolism in the hypothalamus had a rise by ST-36 acupuncture. Those studies demonstrate that ST-36 acupuncture enhances the digestion function and provides wholistic care to promote health, balance, and vitality. 

There are several limitations in this study. A limitation of the study design is that the laser beam spot size is large relative to the anatomical size of hindlimbs of rats. The responses to laser acupuncture treatment may not be specific to ST36 and KI1. Further studies using laser with smaller beam spot size are needed to address the question whether the laser acupuncture treatment at the other body regions or points which are not necessary on the meridians would show the same responses. In addition, stratified laser output levels can be tested to determine a dose-effect threshold and the optimal laser dose parameter. Another limitation of this study is that the experiment is an *in vitro* laboratory research. Because of the low thickness and anatomical location of articular cartilage, monitoring the tissue *in vivo *has been a challenge. Recently, the arthroscopic ultrasound appears to be a promising method for *in vivo* investigation on articular cartilage [4, 20, 35]. The study design can be improved by using* in vivo* arthroscopic ultrasound imaging to evaluate the articular cartilage in larger animals such as dogs. 

In conclusion, the present study used the UBM system to evaluate the alterations in patellar articular cartilage induced by unloading and the therapeutic effects of LA therapy on the cartilage tissue in rats. The results indicate that 28-day unloading induced significant changes in cartilage thickness and URI and insignificant changes in IRC, IBC, and ultrasonographic score. The daily LA therapy improved the acoustic and morphological properties of the cartilage tissue and prevented the cartilage degeneration. This study suggests that LA therapy would be a simple and safe non-pharmacological countermeasure for unloading-induced cartilage degeneration and the UBM system has potential to be a sensitive, specific tool for quantitative assessment of articular cartilage.

## Figures and Tables

**Table 1:** Comparison of ultrasonic characteristics of patellar articular cartilage in three groups.

Parameter	C	T		L		
		Value	T versus C	Value	L versus C	L versus T
URI (*μ*m)	3.97 ± 1.80	6.38 ± 1.58	+60.9%**	5.48 ± 2.56	+38.1%	−14.1%
IRC (dB)	−33.73 ± 3.55	−37.83 ± 5.83	+12.1%	−36.28 ± 5.60	+7.5%	−4.1%
IBC (dB)	−26.92 ± 2.89	−29.97 ± 4.39	+11.3%	−28.91 ± 4.42	+7.4%	−3.5%
Cartilage thickness (*μ*m)	147.00 ± 23.99	126.97 ± 21.12	−13.6%*	140.22 ± 19.61	−4.6%	10.4%
Ultrasonographic grade	2.09 ± 0.54	2.42 ± 0.51	+15.6%	2.20 ± 0.79	+5.2%	−9.0%

*P < 0.05; **P < 0.01.
